# Physiotherapeutische Differenzialdiagnose von Rückenschmerzen im Zusammenhang mit Endometriose

**DOI:** 10.1007/s00482-024-00795-0

**Published:** 2024-02-23

**Authors:** Elisabeth Oberegger, Bernhard Taxer

**Affiliations:** 1 Graz, Österreich; 2 Studiengang PTH, FH JOANNEUM Graz, Eggenberger Allee 13, 8020 Graz, Österreich

**Keywords:** Physiotherapie, Endometriose, Low Back Pain, Management, Differenzialdiagnose, Gynäkologie, Physiotherapy, Endometriosis, Low back pain, Management, Differential diagnosis, Gynecology

## Abstract

**Hintergrund:**

Endometriose (EM) ist eines der häufigsten gynäkologischen Krankheitsbilder in unserer Gesellschaft. Die Diagnose des Krankheitsbilds dauert im Durchschnitt 7 bis 10 Jahre. Um diesen Zeitraum zu verkürzen, muss dieses Syndrom mehr Aufmerksamkeit bekommen. Das Ziel des vorliegenden Artikels ist es, Überschneidungen der beiden Krankheitsbilder EM und Low Back Pain (LBP) zu untersuchen und deren Relevanz für das physiotherapeutische Screening zu beschreiben.

**Fragestellung:**

Welche klinischen Zeichen haben die Syndrome EM und LBP gemein und inwieweit kann das physiotherapeutische Screening gynäkologische Aspekte berücksichtigen und dementsprechend angepasst werden?

**Ergebnisse:**

Um diese Fragestellung zu beantworten, wurden Entstehung sowie Symptome der beiden Syndrome recherchiert und auf Überschneidungen gescreent. Diese wurden dann in Bezug auf die bereits vorhandene Literatur und Fallstudien gesetzt. Die aktuelle Forschungslage zeigt Überschneidungen der beiden Krankheitsbilder hinsichtlich der Ätiologie, des Schmerzmechanismus sowie eines nicht zu unterschätzenden psychosozialen Aspekts. Die Studienlage zeigt, dass mehr Frauen als Männer von LBP und anderen chronischen Schmerzsyndromen betroffen sind. Die EM kommt fast ausschließlich bei Frauen vor und zählt wie der LBP zu den chronischen Schmerzsyndromen. So wird in der Literatur ein gemeinsamer Schmerzmechanismus der beiden Syndrome diskutiert. Die häufigste Überschneidung der beiden Krankheitsbilder zeigt sich durch das Auftreten des LBP als häufiges Symptom der EM, wobei dieser Zusammenhang durch strukturelle Ursachen sowie durch eine reflektorische Schmerzpräsentation begründet werden kann.

**Schlussfolgerung:**

In der Physiotherapie können Hinweise auf EM in der Anamnese und physischen Untersuchung beobachtet werden. Das Berücksichtigen dieser Faktoren kann dabei helfen, die Diagnosezeit der EM zu verkürzen, indem man bei einem Verdacht auf eine gynäkologische Beteiligung bei LBP auf eine weitere Abklärung verweist. Eine umfassende Anamnese ist wichtig und sollte urologische, gynäkologische sowie sexualanamnestische Aspekte abdecken.

## Abstract (en)

**Background:**

Endometriosis (EM) is one of the most common gynecologic conditions in our society. The diagnosis takes an average of 7 to 10 years. To shorten this period, this syndrome needs more attention. The aim of this article is to show overlaps between EM and low back pain (LBP) and to describe their relevance for physiotherapeutic screening.

**Objectives:**

What clinical signs do the syndromes EM and LBP have in common and to what extent can physiotherapeutic screening take gynecological aspects into account and be adapted accordingly?

**Results:**

To answer this question, the two syndromes were screened for overlaps. These overlaps were then related to the existing literature and case studies. The current research situation shows overlaps of the two syndromes with regard to the etiology, the pain mechanism as well as a psychosocial aspect. The literature shows that more women than men are affected by LBP and other chronic pain syndromes. EM occurs almost exclusively in women and, like LBP, is considered a chronic pain syndrome. Thus, a common pain mechanism of the two syndromes is discussed in the literature. The most frequent overlap of the two syndromes is shown by the occurrence of LBP as a frequent symptom of EM. This connection can be justified by structural causes as well as by a reflex pain presentation.

**Conclusion:**

In a physiotherapeutic setting, evidence of EM can be observed in the history and physical examination. Considering these factors may help shorten the diagnosis time of endometriosis by referring for further evaluation if gynecologic involvement is suspected in LBP. A comprehensive history is important and should cover urologic, gynecologic, as well as sexual history.

In this article, the term woman is used to refer to the biological female sex and is not related to individual gender identity. The clinical picture mainly affects women, which is why in the following work, as far as it concerns the people suffering from the disease, it is not used in the opposite sense.

## Einleitung

Auch wenn die Endometriose (EM) häufig als gynäkologisches Krankheitsbild beschrieben wird, wird sie durch ihren Einfluss auf das Immunsystem und den Hormonhaushalt als systemische Erkrankung bezeichnet [13, 53]. Gekennzeichnet ist EM durch eine Anlagerung von gebärmutterschleimhautähnlichem Gewebe außerhalb der Gebärmutterhöhle. Es kommt zu Entzündungen, Vernarbungen und Wucherungen im Körper, welche je nach Lokalisation und Phänotyp zu unterschiedlichen Symptomen führen [2]. Eines der Symptome ist unterer Rückenschmerz, welcher für sich alleinstehend in der physiotherapeutischen Praxis häufig anzutreffen ist.

Neben Symptomen wie Chronic Pelvic Pain (CPP), Rückenschmerzen, Dysmenorrhö, Dyspareunie (Schmerzen im Genitalbereich beim Geschlechtsverkehr) sowie Auffälligkeiten in der Menstruationsblutung treten auch Kopfschmerzen, Übelkeit oder Flankenschmerzen auf. Je nach Lokalisation der Herde ist das Auftreten von Schulter- und Brustschmerzen, blutigem Husten oder Pneumothorax möglich [1, 13, 21].

Viele Betroffene leiden jahrelang unter diesen teilweise starken Schmerzen, welche durch ihr Unwissen über diese Erkrankung von ihnen sowie deren Angehörigen oft einfach hingenommen werden müssen [9]. Durch rechtzeitiges Erkennen spezifischer klinischer Zeichen und die daraus resultierende multiprofessionelle Zusammenarbeit könnte eine raschere Diagnosestellung und adäquate Behandlung von Patientinnen ermöglicht werden [22, 39].

Die Pathogenese der EM ist bis dato unbekannt, kann jedoch von Alter, Ernährung, Einnahme hormoneller Kontrazeptiva, Body-Mass-Index (BMI) und der allgemeinen körperlichen Aktivität beeinflusst werden. Die Diagnose der EM wird bei entsprechender Symptomatik durch klinische vaginale Untersuchungen, bildgebende Faktoren sowie eine diagnostische Laparoskopie gestellt [13].

Die lange vorherrschende Transplantationstheorie nach Sampson (1925) beschreibt die Entstehung peritonealer und ovarieller EM basierend auf einer retrograden Menstruation. Sampson (1927) beschreibt auf Basis seiner Forschung ein mögliches Eintreten kleiner Zellen des Endometriums in die Lymphbahnen, welche sich in Folge im gesamten Körper verteilen [41]. Aktuellere Forschungsansätze zeigen Veränderungen in der Lymphangiogenese bei Frauen mit EM, welche das Eintreten von Endometriosezellen in die Lymphbahn begünstigen könnten [24, 27]. Auch eine Autotraumatisierung des Endometriums wird vermutet, welche durch körpereigene Vorgänge und Funktionen während des Menstruationszyklus oder aufgrund iatrogener Traumata entsteht. Die natürlichen peristaltischen Bewegungen und Kontraktionen des Uterus sind bei EM verstärkt. Dies wurde durch untersuchtes Menstruationsblut von Betroffenen gezeigt, welches Zellen der Basalis, der Schicht der Gebärmutterschleimhaut, welche im Normalfall nicht abgeht, enthielt. Mikroverletzungen in der Muskelschicht der Gebärmutter sowie die Metastasierung der Zellen über den Eileiter in das Peritoneum können Ursprung von Endometrioseherden sein. Dieser permanente Wechsel zwischen Verletzung und Reparatur des Gewebes wird als Tissue-Injury-and-Repair-Mechanismus, kurz TIAR-Mechanismus, bezeichnet [32].

Das Immunsystem scheint, neben hormonellen Dysbalancen, eine tragende Rolle in der Pathogenese von EM zu spielen. Durch Fehlregulationen des Immunsystems treten Entzündungen auf, die hauptursächlich für Zellproliferationen und Infiltrationen sind. Apoptotische Vorgänge werden durch die bestehenden Entzündungen behindert, sodass potenziell schädliche Zellen weitere Herde bilden können [26, 31].

Da eine familiäre Häufung nachweisbar ist, beziehen sich weitere Theorien auf einen möglichen Vererbungsmechanismus, welcher bisher wissenschaftlich jedoch noch nicht bestätigt werden konnte [13].

Entsprechend der Lokalität ihres Auftretens kann man die EM in drei Kategorien teilen [48]:

- Endometriosis genitalis Interna (auch bezeichnet als Adenomyosis uteri bzw. Adenomyose, diese beschreibt die Infiltration in die Muskelschicht der Gebärmutter),
- Endometriosis genitalis externa (Befall außerhalb der Gebärmutter innerhalb des kleinen Beckens; schließt somit die peritoneale, ovarielle und tiefe infiltrierende Endometriose (TIE) mit ein),
- Endometriosis extragenitalis (Vorkommen von Endometrioseherden außerhalb des kleinen Beckens, beispielsweise im Brustraum).

Den Grundstein der Diagnosestellung bilden Anamnese sowie spezifische gynäkologische Untersuchungen, welche die bimanuelle Tastuntersuchung, die Spiegeluntersuchung der hinteren Scheidenwand und des äußeren Muttermunds sowie eine Ultraschalluntersuchung des kleinen Beckens beinhalten. Durch diese nichtinvasiven Methoden können geschulte Gynäkolog:innen bereits einige Arten der EM erkennen. Eine Sicherung der Diagnose gelingt durch Gewebeentnahme mittels Laparoskopie und anschließende Untersuchung des Gewebes [45]. Während dies lange als Goldstandard galt, empfiehlt die aktuelle Leitlinie dieses Vorgehen erst bei negativen Ergebnissen der Bildgebung sowie unzureichendem Therapieerfolg nach nichtinvasiver Diagnosestellung [13].

## Low Back Pain (LBP)

Unterer Rückenschmerz wird als Schmerzen unterhalb der zehnten Rippe und oberhalb des Gesäßes definiert und kann mit Ausstrahlungen in das Bein einhergehen [5]. LBP tritt bei Frauen in den unterschiedlichsten Altersstufen häufiger auf als bei Männern, was je nach Alter an unterschiedlichen Faktoren liegt. Man geht davon aus, dass das erhöhte Vorkommen psychologischer Faktoren, das Auftreten von Hormonschwankungen oder die Menstruation dafür verantwortlich sein könnten. Weiters treten chronische Schmerzsyndrome per se, zu denen der anhaltende LBP zählt, häufiger bei Frauen als bei Männern auf [20, 51].

Spezifische bzw. extravertebrale Ursachen für Kreuzschmerzen, wie Entzündungen der Viszera, oder teilweise auch gefährliche Pathologien treten zu ungefähr 0,5–2 % auf und maskieren sich vor allem prodromal als muskuloskelettale Beschwerden [6, 14, 33]. Nichtspezifische Rückenschmerzen bedeuten, dass es keine nachvollziehbare klare medizinische Ursache für das Auftreten von Rückenschmerz gibt; sie sind in der Regel selbstlimitierend und bei Einhalten der bestehenden Leitlinien gut zu behandeln [5, 16, 36].

## Symptome der Endometriose

Zu den typischen Symptomen der EM zählen die Dysmenorrhö, Dyspareunie, Miktions- und Defäkationsbeschwerden, Infertilität sowie Blutungsstörungen. Des Weiteren können Rückenschmerzen sowie chronische Müdigkeit im Zusammenhang mit der EM auftreten [1].

Die Dysmenorrhö beschreibt die schmerzhafte Regelblutung und wird in eine primäre und eine sekundäre Form unterteilt. Treten die Schmerzen unmittelbar nach der Menarche und ohne weitere Pathologien auf, spricht man von der primären Form. Bei der sekundären Form treten die Schmerzen erst einige Zeit nach der Menarche auf und nehmen an Intensität zu. Die sekundäre Dysmenorrhö tritt häufig bei Personen mit EM, Adenomyose, Myomen und weiteren gynäkologischen Pathologien auf [40]. 77 % aller EM-Betroffenen zeigen eine sekundäre Dysmenorrhö, welche somit das Kardinalsymptom der EM ist [1]. Der Schmerzcharakter ist dumpf, krampfartig und lässt sich von „normalen“ Regelschmerzen klar differenzieren [9].

Als Dyspareunie werden Schmerzen beim Geschlechtsverkehr bezeichnet, welche für viele Frauen besonders belastend sind [9]. Diese treten vor allem bei Befall der sakrouterinen Bänder, des Septums zwischen Rektum und Vagina sowie der Rektumvorderwand auf [2]. Die Schmerzen können während oder nach dem Geschlechtsverkehr auftreten und mitunter für mehrere Tage bestehen bleiben. Vielen Betroffenen ist der Zusammenhang der Dyspareunie mit EM nicht bekannt. Sie passen ihr Sexleben entsprechend an, indem die Häufigkeit reduziert oder Stellungswechsel vorgenommen werden. Diese Beschwerden werden im ärztlichen Gespräch aufgrund eines Schamgefühls oftmals nicht erwähnt [9].

Zyklusabhängige Darm- und Blasenstörungen können durch Infiltrationen in Blase oder Darm ausgelöst werden. Eine Rektuminfiltration kann beispielsweise zur Dyschezie, d. h. Stuhlinkontinenz, führen. Es kommt zu Blähungen, Durchfällen, Magenschmerzen, Völlegefühl oder Krämpfen. Schmerzen beim Harnlassen, die Dysurie, entstehen aufgrund von Verwachsungen um die Blase oder Infiltrationen in diese. Störungen der Blasen- und Darmfunktionen können jedoch auch durch eine vegetative Beteiligung ausgelöst werden [12].

Ein unerfüllter Kinderwunsch betrifft zwischen 25 und 50 % der Betroffenen. Ursächlich für die Unfruchtbarkeit sind einerseits die Verwachsungen, welche zu Destruktionen der reproduktiven Organsysteme führen, andererseits werden durch die Entzündungsherde Zytokine freigesetzt, die wiederum die Fertilität herabsetzen. Therapiemöglichkeiten bei EM-assoziierter Infertilität sind die operative Entfernung der Endometrioseherde sowie Maßnahmen zur medizinisch unterstützten Fortpflanzung [8, 10, 11].

Rückenschmerzen als Symptom der EM können strukturell durch Infiltrationen der Beckenwand, Gefäße, Harnleiter oder des Plexus sacralis verursacht werden [12]. Mit 74 % tritt der LBP bei einer Befragung von 23 Patientinnen mit Ureter-EM als häufigstes EM-spezifisches Symptom auf [35]. Damit einhergehend wird auch die chronische Müdigkeit, das Fatigue Syndrom, als häufiges Symptom beobachtet. Dieses wird auf die vom Körper im Entzündungsprozess freigesetzten Zytokine zurückgeführt [42].

Basierend auf einer Analyse des zeitlichen Ablaufs von Diagnose, Schmerzen, Therapien, Arbeitsleben und sozialen Kontakten von 15 Frauen mit EM kann ein Einblick in das Leben von Betroffenen generiert werden. Der Diagnosezeitraum der Befragten lag im Schnitt bei 6,27 Jahren. Dabei berichten die Frauen, dass ihre Symptome von Ärzt:innen oft als „normale“ Periodenschmerzen „abgewertet“ wurden. Der erlebte Schmerz wird als intensiv, lähmend und überwältigend beschrieben. Bildliche Schmerzbeschreibungen sind sich in die Eierstöcke bohrende Messer oder krallende Fingernägel im Unterbauch. Die Schmerzen treten meist mit Beginn der Menstruation auf, einige Frauen beschreiben allerdings ähnliche Schmerzen während des Eisprungs und wieder andere erleben einen permanenten Schmerz. Ein beträchtlicher Anteil hat Erfahrungen mit Dyspareunie, wobei die Penetration starke Schmerzen verursacht. Stellungswechsel oder Einschränkungen in der Häufigkeit des Geschlechtsverkehrs sind gängige Coping-Mechanismen. Alle Frauen durchlebten unterschiedliche Behandlungsansätze, wobei der operative Ansatz die besten Resultate erzielte. Die verschiedenen medikamentösen Ansätze gehen mit Nebenwirkungen, wie Haarausfall oder verstärkten Blutungen, einher. Häufig kommt es durch die starken Symptome zu Einschränkungen im sozialen Leben. Im Beruf kommt es, durch die Einnahme starker Schmerzmittel und Schmerzen per se, zu eingeschränktem Arbeiten und Arbeitsunfähigkeit. Um aus diesen Beschreibungen auf die Gesamtheit der Betroffenen zu schließen, ist die Anzahl der Befragten zu gering. Letztendlich scheint EM jedoch die Lebensqualität der Frauen stark zu beeinflussen. Im Speziellen sollen Angehörige der Pflege eine wichtige Rolle im Erstkontakt mit Patientinnen einnehmen. Sie haben, auch durch eine vertrauensvolle Beziehung zu den Patientinnen, die Möglichkeit, bei sensiblen Themen wie der Dyspareunie hellhörig zu werden und die Symptome der Frauen ernst zu nehmen [9].

## Schmerzmechanismen in Zusammenhang mit EM

Die EM-spezifischen Schmerzen können auf unterschiedliche Schmerzmechanismen zurückgeführt werden. Diese sind in Tab. 1 dargestellt.

**Tab. 1:** Schmerzmechanismen und deren klinisches Bild

Schmerzmechanismus	Klinische Zeichen	Neurobiologische Mechanismen
*Nozizeptiv*	Myofaszialer Auslöser im Sinne eines reaktiven Hypertonus	Entzündliche Mechanismen, periphere Sensibilisierung [23]
*Neuropathisch*	Sensorische Dysfunktionen, blitzartige bzw. paroxysmale Schmerzen, auftretend z. B. bei Pudendusneuralgie	Ektopische Schrittmacher im betroffenen Nerv, Demyelinisierung aufgrund entzündlicher Vorgänge [7]
*Noziplastisch*	Sekundäre Hyperalgesie, Allodynie, emotionale und funktionelle Beeinträchtigungen	Störung der zentralen Reizweiterleitung, aufrechterhaltende zentrale Sensibilisierung [29]

Neben den als noziplastisch zu beschreibenden Komponenten kann es im Rahmen einer Endometriose auch zu nozizeptiven Stimuli durch myofasziale Auslöser wie etwa bewegungsinitiierte Schmerzen beim Geschlechtsverkehr kommen. Neuropathische Komponenten treten beispielsweise in Form einer Pudendusneuralgie auf und präsentieren sich im Bereich der Vulva.

Grob kann man den Schmerzmechanismus bei EM in periphere und zentrale Ursachen unterteilen. Periphere Einflussfaktoren sind die EM-Herde, das Immunsystem sowie periphere Nervenfasern in den Läsionen, dem benachbarten Peritoneum und peripheren Neuronen. Auf zentraler Ebene haben beispielsweise neurophysiologische Adaptierungen und Stressoren einen Einfluss auf das Schmerzempfinden der Patientin [34].

Einen möglichen Zusammenhang verschiedener chronischer Schmerzsyndrome mit EM, gestützt auf gemeinsame ätiologische und pathophysiologische Mechanismen, beschreibt Häuser (2021) in einer selektiven Literaturrecherche. Der relevanteste gemeinsame Pathomechanismus ist die gestörte zentrale Reizverarbeitung. Die daraus resultierende reduzierte sensorische Schwelle führt zu einer erhöhten Sensitivität gegenüber verschiedenen, auch nicht schmerzhaften, Reizen. Weiters wurden bei Patienten und Patientinnen mit chronisch überlappenden Schmerzsyndromen (COPC) eine reduzierte endogene Hemmung sowie eine Summation bei wiederholten nozizeptiven Reizen festgestellt. Dieser Mechanismus wird als „Wind-up-Phänomen“ bezeichnet und deutet auf eine zentrale Sensibilisierung hin [21].

Zudem ist die Prävalenz einiger chronischer Symptome wie Fatigue, chronische unspezifische Kreuzschmerzen, chronische Spannungskopfschmerzen, Endometriose, Fibromyalgiesyndrom, interstitielle Zystitis, Migräne, Reizdarmsyndrom, temporomandibuläre Dysfunktion (TMD) und Vulvodynie in der weiblichen Bevölkerung höher [21]. Die EM scheint durch Überlappungen mit dem Fibromyalgiesyndrom, Migräne, interstitieller Zystitis, Reizdarmsyndrom und der Vulvodynie zu korrelieren [21].

Patientinnen mit EM sollten daher auf weitere Schmerzsyndrome gescreent werden. Bei der physischen Untersuchung wird auf eine Hyperalgesie und Allodynie geachtet, welche Hinweise auf eine anhaltende zentrale Sensibilisierung sein können. Im Sinne der Schmerzreduktion empfiehlt es sich wahrscheinlich bei diesen Patientinnen wiederholte operative Eingriffe zu vermeiden. An erster Stelle steht eine multimodale Therapie, welche Physiotherapie und eine schmerzmedizinische Behandlung inkludiert [21, 43].

Eine retrospektive Analyse medizinischer Befunde von 138 Probandinnen zeigt ebenfalls einen Zusammenhang zwischen EM und anderen COPCs auf. Die erhobenen Dokumente aus der Gynäkologie, Urologie, Gastroenterologie, Psychiatrie, Neurologie, physikalischer Medizin sowie Familiengeschichte wurden hinsichtlich des Auftretens verschiedener Schmerzsyndrome, Asthma und der Stimmungszustände Angst und Depression gescreent. 56 % der Patientinnen zeigen zumindest eines, 27 % zwei oder mehr COPCs auf. Das am häufigsten auftretende Schmerzsyndrom ist das Reizblasensyndrom, darauf folgen chronische Kopfschmerzen, der LBP und die Zystitis. Zudem zeigt sich bei Patientinnen mit EM ein erhöhtes Aufkommen an Autoimmunerkrankungen. Auffällig ist neben dem Auftreten eines bzw. zweier COPCs die hohe Rate an psychoemotionalen Faktoren [47]. Dies trägt zusätzlich zu einer anhaltenden zentralen Sensibilisierung bei und unterstützt damit den noziplastischen Schmerzmechanismus [15].

## Therapie der EM

Aktuell beschriebene Therapien beziehen sich auf medikamentöse, physisch orientierte, psychologische und komplementäre Methoden, welche in Tab. 2 dargestellt sind.

**Tab. 2:** Therapiemöglichkeiten der EM [2, 13, 17, 18]

Therapie	Ziele	Vorgangsweise
*Medikamentös*	Hemmung der Proliferation des Gewebes Schmerzreduktion	Gestagenmonotherapie (systemisch/lokal mittels Hormonspiralen)
		Analgetika (NSAR, GnRH-Analoga)
*Physisch*	Reduktion der Stressoren zur Entzündungsreduktion Regulation des Kortisolhaushalts	Schulung der Körperwahrnehmung
		Atemübungen
		Stretching
		Aktive und passive Mobilisation der Gelenke in Schmerzregionen
		Beckenbodentraining
		Massage
		Yoga
		Transkutane elektrische Nervenstimulation
		Entspannungstechniken
*Psychologisch*	Reduktion der Stressoren zur Entzündungsreduktion	*Psychotherapeutische Gespräche zu den Themen:*
		– Schmerz, Endometriose
		– Partnerschaft, soziale Kontakte und Familie
		– Sexualität
*Komplementärmedizinische Methoden*	Ergänzend zur schulmedizinischen Therapie Wissenschaftliche Begründung mangelhaft, aber Wirkungen beschrieben	Umweltmedizin
		Orthomolekulare Therapie
		Phytotherapie
		Enzymtherapie
		Mikrobiologische Therapie
		Homöopathie
		Bachblütentherapie
		Akupunktur

*NSAR* nichtsteroidale Antirheumatika, *GnRH* Gonadotropin-Releasing-Hormon

Neben den in der Tabelle dargestellten konservativen Behandlungsmethoden zählt die Laparoskopie bei operativer Indikation zum Standardverfahren [13].

Prinzipiell werden zur Schmerzreduktion initial NSAR (nichtsteroidale Antirheumatika) verabreicht. Aufgrund der Östrogenbeteiligung bei EM ist der Einsatz von Gestagenpräparaten sowie hormonellen Kontrazeptiva zum Unterdrücken der Ovulation angezeigt. Bei entsprechendem Leidensdruck oder ausbleibendem Erfolg durch die medikamentöse Therapie wird mittels operativer Sanierung therapiert. Je nach Ausprägung und EM-Befall sowie der Lebenssituation der Patientin kann diese durch Entfernung des EM-Gewebes oder durch (Teil‑)Resektion betroffener Organe ausfallen. Bei der operativen Therapie muss der Fertilitätserhalt, in Rücksprache mit den Betroffenen, beachtet werden. Bei unerfülltem Kinderwunsch nach operativer Intervention können zusätzlich reproduktionsmedizinische Interventionen eingesetzt werden. Als Rezidivprophylaxe wird nach erfolgter EM-Operation eine weitere medikamentöse Therapie empfohlen. Ein interdisziplinäres und individuelles Vorgehen in Diagnostik und Therapie ist notwendig, um die optimale Therapie für jeden Fall zu gewährleisten [1].

## Differenzialdiagnose in der Physiotherapie

Im Zuge der physiotherapeutischen Untersuchung ist es unumgänglich, neben einem strukturell-funktionellen und einem psychosozialen (Yellow Flags) Screening auch spezifisch medizinische und teilweise auch dementsprechend gefährliche (Red Flags) Aspekte zu bedenken, die sich als neuromuskuloskelettale Symptome maskieren könnten und somit differenzialdiagnostisch zu bedenken sind [3, 30]. In der physiotherapeutischen Praxis können unterschiedliche Zeichen und Symptome bereits in der subjektiven und objektiven Untersuchung darauf hindeuten und nach weiterer Abklärung verlangen. Endometriosezysten, Verwachsungen und Infiltrationen im kleinen Becken sowie Befall der Wirbelkörper stellen eine Ursache für muskuloskelettale Beschwerden wie LBP dar [8, 11, 38]. Wie der Tab. 3 zu entnehmen ist, können Schmerzcharakter, Schmerzlokalisation sowie begleitende Symptome erste Hinweise enthalten.

**Tab. 3:** Symptomübersicht der Fallbeispiele

Fallstudie	Symptome	Schmerzlokalisation	Schmerzcharakter	Diagnose
*Piekartz (2012)* [38]	LBP (2) Leistenschmerz (1) Variable Zyklusdauer Fatigue 2 W/M Druck im Unterbauch beim GV und langem Sitzen	Leiste li (1) LWS ausstrahlend in die li. lat. Gesäßhälfte (2)	Diffus, krampfartig (1) Tief, diffus, dumpf (2) Auftreten sporadisch u. während Menstruation	Ovarielle Endometriose mit Zyste am Ovar
*Cricco et al. (2021)* [8]	LBP Dysmenorrhoe Dyspareunie	LWS m. Ausstrahlung in Leiste li u. li Sitzbeinhöcker	Konstant im 24-h-Verhalten – in Ruhe Schmerzen NPRS 7/10	Endometriosezyste am Ovar mit Beteiligung des uterosakralen Ligaments
*Dongxu et al. (2014) *[11]	LBP	LWS	Menstruationsabhängig	Spinale Endometriose mit Infiltration des LWK 3

*LBP* Low Back Pain,* LWS* Lendenwirbelsäule, *LWK* Lendenwirbelkörper, *(1)/(2)* Symptomzuordnung, *W/M* Wochen pro Monat, *li* links, *lat.* lateral, *NPRS* Numeric Pain Rating Scale, *−* Reduktion, *GV* Geschlechtsverkehr

Die Autor:innen der recherchierten Fallberichte gaben entweder nach der subjektiven Untersuchung, der physiotherapeutischen Untersuchung oder nach den ersten Therapien die Empfehlung einer weiteren fachspezifischen Untersuchung ab. Gemeinsamkeit der dargestellten Fallstudien ist dabei das Auftreten menstruationsabhängiger Symptome. In allen Fällen wurde eine EM festgestellt (siehe Tab. 3). Dies zeigt die hohe Sensitivität und das Potenzial von Therapeut:innen auf, durch spezifische physiotherapeutische Untersuchungen zur rascheren Diagnosestellung und somit zur zielorientierten Therapie beizutragen.

Das hohe Aufkommen unspezifischer Rückenschmerzen bei Frauen in der physiotherapeutischen Praxis rechtfertigt die Dringlichkeit eines konsequenten Clinical-Reasoning(CR)-Prozesses. Dieser beinhaltet ein permanentes Reevaluieren der eigenen Maßnahmen sowie das Erkennen möglicher spezifischer Faktoren in der subjektiven Untersuchung und dem weiteren Behandlungsablauf. Die Literatur beschreibt noch keine klaren Kriterien, die es Physiotherapeut:innen möglich machen, LBP-Patientinnen genauer hinsichtlich EM zu screenen. EM muss jedoch bei Frauen mit unspezifischem, persistierendem LBP und Beckenschmerz stets als Auslöser in Betracht gezogen werden [25].

Der physiotherapeutische Prozess besteht aus drei übergeordneten Phasen: der Problemidentifizierung, der Planungs- und der Umsetzungsphase. Die Problemidentifizerungsphase besteht aus Anamnese bzw. subjektiver Untersuchung, Datenbeschaffung und Erstellung des physiotherapeutischen Befunds. Dies impliziert Inspektion sowie gezielte strukturelle und funktionelle neuromuskuloskelettale Testungen, welche die Funktionalität der betroffenen Strukturen prüfen. Basierend auf der physiotherapeutischen Diagnose wird das Therapieziel definiert und ein Behandlungsplan erstellt. Die ständige Reevaluierung des Therapieerfolgs und eventuelle Anpassung des Behandlungsplans ist notwendig, um einen erfolgreichen Therapieabschluss zu gewährleisten [37].

**Abb. 1:**
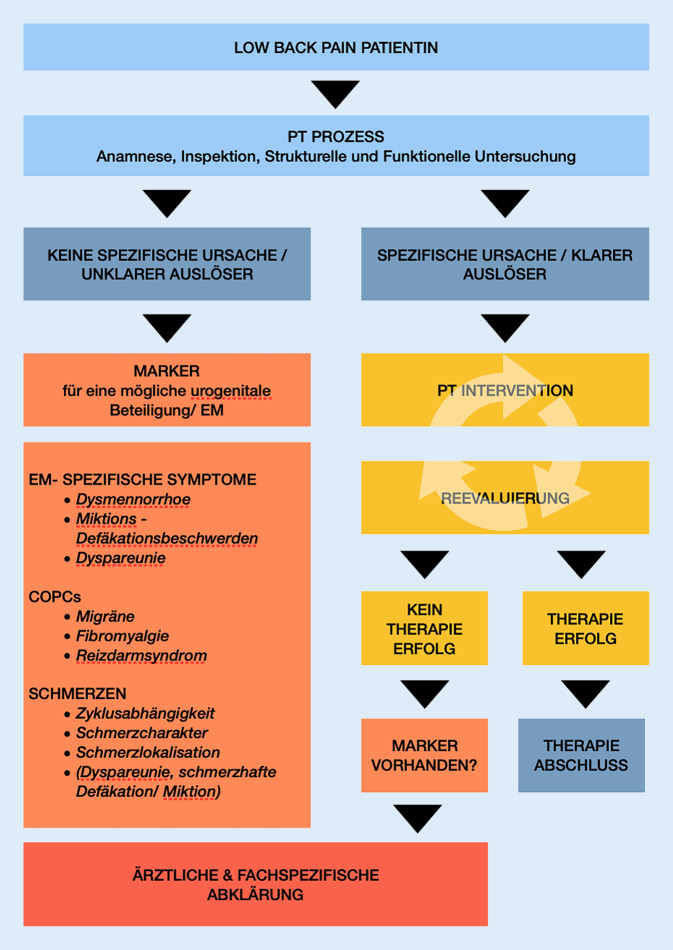
Hinweise auf Endometriose in der PT. Mit Daten aus [8, 11, 21, 38]

Abb. 1 fasst die Erkenntnisse der Recherche dieser Arbeit zusammen und implementiert diese in Form eines physiotherapeutischen Denkprozesses. Sie soll ein Werkzeug für die Praxis darstellen, um Patientinnen auf EM zu screenen, und somit zur früheren Diagnostik dieses Krankheitsbilds beitragen.

Die genannten Faktoren sind Marker, welche bei Patientinnen mit LBP unklarer Herkunft sowie bei unauffälliger oder unschlüssiger Anamnese und physiotherapeutischer Untersuchung auf eine EM hinweisen können. Kommt es während der subjektiven Untersuchung oder im Laufe des physiotherapeutischen Prozesses zu Auffälligkeiten in den gelisteten Bereichen, so ist eine weitere fachärztliche Abklärung notwendig.

Sexualität ist ein fundamentaler Bestandteil des Lebens, daher sollte eine kurze Sexualanamnese in die Routineanamnese mitaufgenommen werden [52]. Medizinische Fachkräfte, wie Physiotherapeut:innen, haben das Potenzial, diesen Teilbereich zu normalisieren und somit schrittweise zu enttabuisieren [49]. Zudem kann eine gezielte Sexualanamnese Aufschluss über etwaige Schmerzzustände geben und somit ausschlaggebend für den weiteren Therapieverlauf sein [44]. Berichtet eine Patientin mit LBP beispielsweise von Schmerzen beim Geschlechtsverkehr, so kann das ein Hinweis in Richtung der EM und anderer gynäkologischer Veränderungen sein. Tab. 4 listet Fragen auf, die bei Patientinnen mit unterem Rückenschmerz inklusive Verdacht auf EM Aufschluss geben können. Weitere Tools für eine gezielte Sexualanamnese sind der deutsche Beckenbodenfragebogen sowie der Fragebogen für viszerale und urogenitale Schmerzen bei Frauen [19, 36].

**Tab. 4:** Fragestellung im Zuge der subjektiven Untersuchung

*Allgemeine Fragestellungen*	Sind Sie mit Ihrem Sexleben zufrieden? [36]
	Haben Sie Schmerzen während oder nach dem Geschlechtsverkehr?
*Spezifische Fragestellungen*	Fühlen Sie sich in Ihrem Sexleben durch ihre Schmerzen eingeschränkt?
	Können Sie Unterschiede bei Stellungswechseln festmachen?
	Wie gehen Sie mit den Schmerzen um?

Mit Hilfe der allgemeinen Fragestellungen können sich Therapeut:innen an das Thema herantasten und den Patientinnen Raum geben, von sich aus zu berichten. Erleben die Patientinnen keine Einschränkungen in ihrem Sexualleben, ist eine weitere Anamnese vorerst nicht relevant. Die spezifischeren Fragen können einerseits Aufschluss über den psychosozialen Zustand der Betroffenen geben, andererseits bieten sie Raum für die Überlegung zu diversen Coping-Strategien, wie beispielsweise Stellungswechsel bei mechanischen Schmerzauslösern.

## Diskussion

LBP scheint ein häufiges Symptom bei EM zu sein, welches im Zuge des Clinical-Reasoning-Prozesses in der Physiotherapie beachtet werden muss. Die beiden Krankheitsbilder zeigen Überschneidungen bezüglich Prävalenz, Ätiologie, Pathomechanismus sowie Symptomatik. Als häufig auftretendes Symptom ist der LBP, je mehr Überschneidungen es zur Symptomatik der EM gibt, durch eben diese bedingt.

Der Pathomechanismus der EM ist noch nicht eindeutig geklärt, es zeigt sich jedoch ein Zusammenhang zwischen EM und anderen COPCs durch einen gemeinsamen Pathomechanismus. Häuser [21] zählt das Fibromyalgiesyndrom, die Vulvodynie, die Reizblase sowie die Migräne zu den Syndromen, die mit der EM überlappen. Im Sinne eines zentral bedingt erhöhten Schmerzempfindens kann dabei auch die graue Substanz in Thalamus, Putamen und Gyrus cinguli abnehmen [21]. Diese Veränderungen könnte man bei Frauen mit diagnostizierter EM, LBP und anderen COPCs im Vergleich zu Frauen mit diagnostizierter EM ohne begleitende Schmerzsyndrome untersuchen, um eine klare Aussage zu dieser Hypothese treffen zu können.

Die verschiedenen Arten der EM führen zu Herden an unterschiedlichen Stellen im Körper und können so strukturelle Auslöser für LBP sein. Bei der TIE können beispielsweise die sakrouterinen Bänder betroffen sein, bei peritonealer EM können Herde an den Beckenwänden, der Blase, im Douglas-Raum und ebenfalls an den sakrouterinen Bändern auftreten. Die sakrouterinen Bänder entspringen an der Cervix und verlaufen dorsal hin zum Kreuzbein. Die Harnleiter entspringen am Nierenbecken und gliedern sich in einen abdominalen, einen pelvikalen und intramuralen Abschnitt. Sie verlaufen im Retroperitonealraum, überkreuzen im Bereich des kleinen Beckens den Harnleiter sowie bei Frauen die Arteria uterina. Durch eine Verwachsung des Ureters im Bereich des kleinen Beckens ist eine Beteiligung der sakrouterinen Bänder wahrscheinlich, sodass eine Beeinflussung des Sakrums durch diese möglich ist. Demnach könnte das Sakrum Quelle einer strukturellen Ursache des LBP sein [12].

Die ovarielle EM ist durch Zysten an den Eierstöcken charakterisiert. Diese sogenannten „Schokoladenzysten“ enthalten altes Menstruationsblut. Bei der TIE kommt es zu retroperitonealen Infiltrationen, welche teils organdestruierend sein können. In beiden Fällen kann es zur Bedrängung des Ursprungsgewebes kommen, sodass dessen Funktionen stark beeinträchtigt werden. Eine Einengung des Harnleiters kann beispielsweise zu einem Harnaufstau oder zur Sterilität durch Deformation der Eileiter und Eierstöcke führen [2]. Diese pathoanatomische Veränderung ist ein Erklärungsmodell für die Dyspareunie und kann ebenso dem Auftreten des LBP als Symptom der EM zugeschrieben werden [47].

Der LBP bei EM könnte auch durch reflektorische Schmerzpräsentationen begründet werden. Bestimmte Hautareale der inneren Organe werden weiter außen liegenden Schichten aufgrund der nervalen Innervation desselben Segments zugeschrieben und geben Aufschluss über viszerale Beteiligung bei Schmerzen. Sogenannte Head-Zonen für die Beckenorgane liegen dabei im Bereich der Lendenwirbelsäule (LWS) [19].

Psychosoziale Aspekte haben einen nennenswerten Einfluss auf LBP [4, 28, 46, 50]. Betrachtet man die Erfahrungsberichte von Patientinnen mit EM, kann man auch hier Faktoren erkennen, die das Krankheitsbild und Erleben der Krankheit beeinflussen. Die Symptome der EM sind in unserer Gesellschaft, trotz des zunehmenden Bewusstseins, immer noch ein Tabuthema. Monatliche Arbeitsausfälle aufgrund starker Schmerzen und unzureichende Kenntnisnahme durch behandelnde Ärztinnen und Ärzte, welche die Schmerzen als übliche Periodenschmerzen abwerten, sind beitragende Faktoren auf psychosozialer Ebene. Zusätzlich sind Partnerschaft bzw. das Sexualleben betroffener Frauen durch die Dyspareunie maßgeblich eingeschränkt [9].

Die durchschnittliche Diagnosestellung der EM dauert 7 bis 10 Jahre. LBP stellt als Überlappungssyndrom eine Art Schnittstelle zwischen der allgemeinen physiotherapeutischen Tätigkeit und der spezifischen Erkennung und Behandlung von EM dar. Setzt man im physiotherapeutischen Prozess an und berücksichtigt gewisse Faktoren oder Hinweise, die einen Verdacht auf dieses Krankheitsbild darstellen, so können Physiotherapeutinnen und Physiotherapeuten dazu beitragen, diese Diagnosezeit zu verkürzen.

Die EM per se wird hormonell oder operativ behandelt. Eine physiotherapeutische Begleitung kann einerseits begleitende Symptome auf muskuloskelettaler Ebene beeinflussen und andererseits die betroffenen Frauen auf psychosozialer Ebene begleiten. Es ist wichtig, dieses chronische Schmerzbild zu erkennen und den Frauen Beachtung hinsichtlich ihrer Symptomatik zu geben, da viele Frauen hinsichtlich des Ausmaßes ihrer Schmerzen oft über Jahre hinweg nicht wahrgenommen werden. Um derartige psychosoziale Faktoren zu berücksichtigen, erscheint eine multimodale Auseinandersetzung sowohl im Screening als auch in der Behandlung unumgänglich [43].

## Fazit für die Praxis

Diese Arbeit soll einen Beitrag zur schnelleren Diagnosestellung bei Patientinnen mit EM leisten. Der Blick von Physiotherapeutinnen und Physiotherapeuten soll dahingehend geschärft werden, beim Auftreten spezifischer Symptome eine weitere internistische oder gynäkologische Abklärung zu empfehlen. Die Studienlage zeigt verschiedene Zusammenhänge der Krankheitsbilder LBP und EM auf.

*Therapeutische Relevanz:*

- Eine gezielte subjektive Untersuchung durch Therapeutinnen und Therapeuten hinsichtlich der EM als möglichem Auslöser des LBP erscheint als klinisch hochrelevant.
- Eine Sexualanamnese sollte in den physiotherapeutischen Alltag inkludiert werden.
- Neben einer erweiterten Erforschung der EM per se zeigt sich auch im Bereich der Sexualanamnese in der Physiotherapie, der Beachtung gynäkologischer Krankheitsbilder im physiotherapeutischen Setting und hinsichtlich der Zusammenhänge von muskuloskelettalen Beschwerden mit internistischen Krankheitsbildern weiterer Forschungsbedarf.
